# “We are used to this”: a qualitative assessment of the perceptions of and attitudes towards air pollution amongst slum residents in Nairobi

**DOI:** 10.1186/1471-2458-14-226

**Published:** 2014-03-05

**Authors:** Kanyiva Muindi, Thaddaeus Egondi, Elizabeth Kimani-Murage, Joacim Rocklov, Nawi Ng

**Affiliations:** 1 African Population and Health Research Center (APHRC), Nairobi, Kenya; 2 Epidemiology and Global Health Unit, Umeå University, SE-901 85 Umeå, Sweden

**Keywords:** Air pollution, Perceptions, Attitudes, Nairobi, Slums

## Abstract

**Background:**

People’s perceptions of and attitudes towards pollution are critical for reducing exposure among people and can also influence the response to interventions that are aimed at encouraging behaviour change. This study assessed the perceptions and attitudes of residents in two slums in Nairobi regarding air pollution.

**Methods:**

We conducted focus group discussions with residents aged 18 years and above using an emergent design in the formulation of the study guide. A thematic approach was used in data analysis.

**Results:**

The discussions revealed that the two communities experience air pollution arising mainly from industries and dump sites. There was an apparent disconnect between knowledge and practice, with individuals engaging in practices that placed them at high risk of exposure to air pollution. Residents appear to have rationalized the situation in which they live in and were resigned to these conditions. Consequently, they expressed lack of agency in addressing prevalent air pollution within their communities.

**Conclusions:**

Community-wide education on air pollution and related health effects together with the measures needed to reduce exposure to air pollution are necessary towards reducing air pollution impacts. A similar city-wide study is recommended to enable comparison of perceptions along socio-economic groups and neighbourhoods.

## Background

Urban air pollution remains a major health risk to millions of urban residents worldwide as it is estimated that about 1.3 million deaths annually are attributable to urban air pollution [1]. The problem is intensifying in many cities and towns in the developing world where growing urban populations and the attendant increase in activities which have led to a rise in air polluting emissions. This is coupled with industrial growth amidst weak or non-existent environmental protection laws, leading to levels of air pollution that often exceed emissions standards set by the World Health Organization [2-5]. In addition, urban poor populations are more disadvantaged in terms of exposure to air pollution because of poor housing structures, close proximity to air pollution sources and the types of fuel used for cooking. However, little is known about how people view air pollution in urban areas.

Studies on air pollution in Africa and other low- and middle-income countries (LMICs) are few [5]. As LMICs work towards gaining industrialised status, economic growth is emphasized at the expense of the environment and the health of the people. In these contexts, there is greater need for environmental stakeholders to address air [environmental] pollution in order to set the stage for inclusive and well-informed interventions aimed at reducing air [environmental] pollution effect. A starting point for such enquiry would be looking at individual perceptions and attitudes towards air pollution. Gaining knowledge of peoples’ perceptions of air pollution is important, as it reflects the social dimensions and circumstances under which people understand pollution [6]. This knowledge helps to ensure that policy and communication frameworks achieve desired change in public attitudes and behaviour.

To involve lay opinions in the process of policy making, it would be important to understand public perceptions as they are important factors in the successful implementation of environmental policies [6,7]. First, understanding public perceptions of air pollution and associated risk will ensure there is a consultative process in the formulation of policies as opposed to the existing top-down approach where the public is largely a recipient of policy actions without giving any input in their formulation [7]. Secondly, perceptions of pollution and the associated consequences will give implementing agencies the opportunity to assess public knowledge regarding air pollution as well as any misconceptions that might exist. Addressing these misconceptions could increase involvement of the public in policy implementation [5]. Thirdly, understanding people’s perceptions would inform the entry point of actions/interventions aimed at mitigating pollution-related risks [8].

In order to address the prevailing pollution, policies need to be formulated and implemented to curb emissions and encourage air friendly practices among the populace. The acceptance of formulated policies requires the cooperation of the populace as some of the policy actions would impact them directly or may require individual behaviour change. Indeed, the 1992 Earth Summit recommended the inclusion of the public in the environment policy process to ensure the effective implementation of formulated policies [9]. However, there has been little progress on inclusion of public globally as most environmental policies tend to lean more on science than on public opinions [7]. The top-down approach in policy formulation and implementation has been faced with serious challenges as the public view of risk does not overlay the scientific opinion of that risk [10,11]. This disconnect has led to failure of sections of the public adhering to existing policies. For example, a study in a province in China found that the population was largely ignorant of existing environmental protection policies and were not adhering to expected actions [12].

Perception of risk or vulnerability is a central component of health promotion theories and has been found to be shaped by several factors. This study is anchored on the general protection motivation theory [13] that posits that the intention to protect one-self is dependent on: 1) the perceived severity of a threatening event, 2) the perceived probability of the occurrence, or vulnerability, 3) the efficacy of the recommended preventive behaviour, and 4) the perceived self-efficacy to undertake the recommended preventive behaviour. In this study, the perceptions about air pollution levels and related health risks form the first and second components of the theory. The third component refers to the common practices among the residents that are expected to reduce air pollution and exposure levels. Lastly, the perceptions of residents regarding their role in addressing the prevailing air pollution represent the fourth component of the theory. Studies have found that perceptions on levels of air pollution are shaped by the presence of suspected sources of pollution such as industries or busy roads [8,14]. Further, social interactions that ensure the diffusion of knowledge have been shown to shape perceptions [15].

Studies in developed countries show that people in poor neighbourhoods are less likely to report air pollution as an issue of top concern [14]. However, there exist contrary findings in similar contexts [16]. Research evidence suggests that people’s level of attachment to the place of residence and social capital determines how they responded about the levels of air pollution and their willingness to take action against pollution. People with high attachment to their place of residence and those with high social capital are less likely to report air pollution as an issue to avoid stigma, and are also likely to take action to address air pollution [17,18].

In slum areas around the world, environmental degradation ranks among the key challenges residents face [19,20]. This is compounded by the fact that many slums are located near industrial districts or close to busy highways. In addition, crowding, a characteristic of many slums, renders the adoption of measures to reduce pollution at individual household level ineffective due to a ‘neighbourhood effect’ in which adopting households may continue to suffer due to exposure from non-compliant households. Lastly, the political, social and economic exclusion of slum areas [21] puts them in a vulnerable position as they lack systems to manage such things as waste collection or find a collective voice to bargain for services and protection against external polluters such as industries.

There is limited evidence on people’s perceptions and attitudes towards air pollution in Kenya. Therefore, the objective of the study was to assess the perceptions and attitudes of slum residents about air pollution. This study is expected to provide insights into people’s perceptions on air pollution and what they consider to be their role in addressing air pollution. The results emerging from this study will be important in informing other larger studies in similar and/or different contexts as well as informing the design of quantitative studies on air pollution. The results will also be crucial to informing acceptable entry points for interventions to mitigate pollution.

## Methods

### Context

The study was conducted in two slums in Nairobi city; Korogocho and Viwandani. These sites were selected because of an on-going health and demographic surveillance initiated in 2002 which is a reliable sampling frame [22]. A detailed description of the Health and Demographic Surveillance System carried out in the two areas is given elsewhere [22]. One striking difference between the two slums is the proximity of Viwandani to the industrial area where diverse manufacturing activities occur and where traffic flow is constant as trucks deliver materials and pick up finished goods. Alternatively, Korogocho is near the city’s municipal dumpsite and faces several environmental issues arising from proximity to this location. The two slums are also characterized by activities believed to raise the levels of both indoor and outdoor air pollution.

The two sites have some differences regarding the population structure, education and income generating activities. Viwandani residents are more educated and dependent on economic activities that are more stable, while in Korogocho, unstable economic activities dominate. Other differences can be seen in the physical structures of the houses with Korogocho having mostly mud-walled houses with zinc sheet roofing and mud flooring, while in Viwandani, the walls and roofs are made of zinc sheets and floors are cemented. Thus, Viwandani has a better household socio-economic outlook as compared to Korogocho.

In spite of these differences, the two sites are similar in that they are both slum communities located in close proximity to major pollution sources and face similar environmental challenges.

### Design of study and data

We designed a qualitative study on the perceptions and attitudes towards air pollution of people living in Korogocho and Viwandani. This study was designed as an exploratory study in a context of non-existent data on people’s perceptions and attitudes towards air pollution, nor data on levels of air pollutants. The study sought to inform an on-going qualitative study in the same communities as well as inform the monitoring of pollutant levels both at the community level and in individual households.

A total of eight focus group discussions (FGDs) were held with adult residents of the two communities, four in each community. The discussions were separately held with younger adults (18–29 years; n = 39) and older adults (30 and above; n = 42) as it was felt that the younger adults might be intimidated by the presence of older participants, affecting their contribution to the discussions. In addition, youths in slum settings use a colloquial language called “sheng” that is not widely understood by older people. The participants were of mixed gender as there was no anticipated personal information that would be withheld in the presence of the opposite sex. Groups had between nine and 11 participants with roughly equal representation of the sexes. Participants differed in their ethnic background as well as level of education, employment status and duration of stay in the community. This was preferred to ensure a diversity of opinions regarding air pollution. Table 1 summarizes some of the background characteristics of the participants (see Table 1).

**Table 1:** Distribution of FGD participants by select background characteristics

	**Percent**
**Sex**	
Female	51.9
Male	48.2
**Slum of residence**	
Korogocho	49.4
Viwandani	50.6
**Age**	
19-29 years	48.2
30+ years	51.9
**Duration of stay in slum**	
<10 years	28.4
10-19 years	22.2
>20 years	49.4
**Level of education**	
None	12.4
Primary	50.6
Secondary or Higher	37.0
**Occupation Type**	
None	27.2
Business (petty or established)	44.4
Employee (casual or long term)	28.4
**N = 81**	

We employed an emergent design in which we analysed the data and revised the study guide based on results from the first set of discussions. The discussions were therefore conducted in two waves to allow the researchers, time to conduct some analysis of the collected data in order to revise the guide as necessary. In the first round of the FGDs, two groups were convened at each site during the month of November 2012. The second round of discussions was conducted in January 2013. With the help of community mobilizers, the researchers purposively selected the participants. The participants were selected as much as possible from all villages within each slum to ensure that different areas of the slums were represented.

The discussions were conducted in Kiswahili, which is the national language widely spoken in Kenya, and particularly, in the urban slums and well understood by all participants. However, participants were allowed to express themselves in Kiswahili, English or “Sheng” (a mix of Kiswahili, English and local languages). The FGDs were moderated by the second author while the first author took notes. In Korogocho, we conducted the first discussions in an office within a health facility. The second round of discussions was held within an office block a distance away from the health facility where there were fewer disturbances from those visiting or working at the facility. In Viwandani, all discussions were held in a community hall.

On average, the FGDs took 50 minutes and were recorded and later transcribed and translated into English by the note taker. Upon analysis of the first round of discussions (four FGDs), the FGD guide was revised, dropping some of the questions that were not eliciting any new information from the groups and adding new questions that occurred during the earlier discussions. The initial FGD guide had six questions ranging from what comes to the mind of participants when they heard about environmental pollution to issues about the sources of outdoor and indoor air pollution. Furthermore, participants were asked about their thoughts on the government’s and residents’ responsibilities in addressing air pollution in their communities. After revision, the questions on people’s understanding of the environment and risk were dropped as the responses showed no variation in the first four groups. In addition, new questions on participants’ concerns about air pollution and a question to compare perceived levels of indoor and outdoor air pollution were included.

The transcribed discussions were transferred to NVivo 9 to help organise the data for analysis and interpretation. Coding was done based on recurring themes that were identified through reading of transcripts or observations of recurrent issues raised by participants. Other themes were identified based on diversity of views and contradiction. Some broad themes identified through review of literature formed the basis of the interview guide prior to the discussions. Some of these themes were retained in the final analysis while others were refined or new ones formed based on participants’ responses. This was achieved during the transcription process as well as through further reading of the transcribed discussions and analysis of the coded data which done by the lead author. The transcripts were shared with two of the co-authors and a qualitative researcher in Nairobi who is not a co-author but who gave insights into the analysis of the data. However, triangulation of these analyses was not done. Thematic analysis was used because of its appropriateness in selecting the most recurrent perceptions.

### Ethical considerations

The study was reviewed and granted ethical clearance by the African Medical Research Foundation’s (AMREF) ethics review committee. Informed consent was sought in two stages. First, during recruitment, the researchers provided the potential participants with full disclosure regarding the study, which included information on the purpose of the study and procedures. Second, consent was obtained before recording the discussions. Written informed consent was sought for participation and for audio recording just before the discussions started. All participants wore numbered tags which were used as identifiers during the discussions. Participants were informed of the use of number tags as opposed to their actual names in the discussions. In addition they were informed that their identity would not be disclosed in any reports or publications arising from the data.

## Results

The results from the narratives of adult participants from both communities formed seven thematic areas. These included mixed knowledge about sources of air pollution; sensing air pollution; who is to blame?; poor housing and neighbourhood effects; desperate practices; resistance and ignorance; and fatalism and helplessness. The results are structured into sections according to the seven thematic areas identified.

### Knowledge on sources of air pollution

#### Outdoor air pollution

##### Mixed knowledge about sources of air pollution

We sought to learn about participants’ thoughts regarding the sources of outdoor and indoor air pollution in their communities. It emerged from the discussions that residents had mixed knowledge about the sources of air pollution as well as some of the consequences of exposure to this pollution. While participants generally correctly identified sources of outdoor air pollution, there were occasions when it was evident that the knowledge prevailing in these communities was flawed. For example, the view that smelly drainage channels and toilets were a source of air pollution was frequently expressed by both the young and older participants from both communities. Similarly, participants from both communities raised the issue of drainage channels as important sources of air pollution. They were said to emit foul smells due to stagnant water and people’s habits of dumping waste including faecal matter into the channels. Other opinions expressed by the participants was that lack of toilets also led to air pollution as open defecation and the use of ‘flying’ toilets were used as alternatives, as was the common method of emptying pit latrines using uncovered drums - exposing residents to fouls smells. In addition, poverty and preference for cheap fuel materials were mentioned as factors contributing to the use of alternative fuels such as plastic bags, gunny bags, and cloth rags, especially among roadside food vendors in Korogocho, and as such, contributing to air pollution.

##### Sensing air pollution

During the discussions, it was apparent that participants relied more on their senses to assess their exposure to pollution and in identifying sources of pollution. Sensory perception of pollution sources was stressed throughout the discussions with participants using terms such as seeing a cloud of smoke covering the area, pungent smells from factories and dumpsites and soot falling on people and buildings. This brings to the fore the apparent reliance on the senses to inform perceptions on sources and individual exposure.

##### Who is to blame?

There were mixed opinions towards who was responsible for air pollution in the community with some attributing the state of outdoor air to residents while others felt it was primarily due to sources external to the communities. Several participants from Korogocho mentioned the municipal garbage dumpsite as the biggest polluter. The participants mentioned that the dumpsite was always on fire, covering the entire community of Korogocho in smoke that was ‘corrosive’ as the following quote indicates:

That dumpsite is the biggest air pollutant, because when that dumpsite is lit, there is a lot of smoke coming here, it is dark, people cough and there are schools down there [near the dumpsite] sometimes teachers tell the pupils ‘today there has been a lot of smoke so when you go home tell your parents to give you milk’ … it is bad smoke, like acid; there are medicines and many chemicals burning in the dumpsite (older female, Korogocho).

In addition, the burning of medical waste in the open by local clinics, cigarette smoking, dust and motorcycle fumes were also mentioned as major sources of air pollution. Industrial emissions were also mentioned as sources of pollution but these were not seen as important polluters in Korogocho.

Conversely, discussions in Viwandani revealed that industries were perceived as the biggest sources of air pollution. Both the young and older adults were very emotive when discussing the industries’ contribution to air pollution. Sometimes the emissions from the industries were said to be so bad that people had to step far away from the community in order to get fresh air. The following excerpts capture Viwandani residents’ views on the role of industries in air pollution.

…these industries when night comes they start working, they emit smoke [expression of annoyance] even when you are sleeping you just have to wake up … to open the door and stand outside; they emit smoke! [Expression of annoyance]” (young male, Viwandani).

There is an industry here eh; like yesterday they were releasing some chemical eh yesterday, we couldn’t stay up here, those of us who stay up there we couldn’t stay there we had to go elsewhere; you would see people moving away (young male, Viwandani).

In addition, an emerging unofficial dumpsite and people’s habits of burning trash were indicated as other major sources of air pollution in Viwandani. During one of the discussion sessions, participants shared that their community is sandwiched between two major sources of pollution, the industries on one side and the illegal dumping site on the other. Further, participants shared that most of the smoke from the dumpsite was experienced at night when the garbage was burned. Other important sources of air pollution identified were burning of tyres to extract metal and cigarette smoking. It was surprising that as much as people in Viwandani were exposed to vehicular emissions from busy traffic coming in and out of the industrial area, this was not mentioned in any of the discussions.

#### Indoor air pollution

##### Poor housing and neighbourhood effect

With regard to people’s knowledge of indoor air pollution, participants were aware of the sources contributing to the poor quality of indoor air. These ranged from the type of cooking fuel, cooking stoves and smoking of cigarettes indoors. Other sources include the poorly constructed and congested houses in the two communities that were said to encourage cross-pollution from the outdoors and neighbours. Congestion was seen as limiting the use of corridors as cooking points instead of cooking in the same room where the family slept. It emerged that the kerosene used by many households was considered too smoky, and therefore, dangerous to the residents’ health. In addition, poverty was said to promote the use of poor quality second-hand cooking stoves (i.e., stoves that had previously been used) that were seen to emit more smoke as compared with newer ones. In both communities, participants shared that many people used plastic or rubber materials as well as old foam mattresses to light their charcoal stoves, emitting foul smelling smoke. In addition, the small rooms that households occupied were said to magnify the problem of indoor air pollution due to crowding and lack of vents to release bad indoor air; as the following excerpt indicates:

… because houses are not well ventilated and one room serves as the kitchen, bedroom and sitting room and you use a charcoal stove. I have a child who has asthma and I took him to the hospital to get oxygen… they advised me not to cook while the child is in the house. But if there is only one room, it is a must the child is present when cooking is going on… if the houses were proper it would reduce the smoke (Older female, Korogocho).

The discussions revealed the existence of a ‘neighbourhood effect’ that was acknowledged to contribute to indoor air pollution in people’s homes. For example, participants mentioned that people took advantage of the poor structures they lived in and opted to not ventilate their houses when a stove was being used. Instead, they chose to create venting holes on the side of the neighbour’s house, who then bears the brunt of their emissions. Outdoor influences, especially industry emissions and roadside cooking spots were mentioned as important contributors to indoor air pollution.

##### Desperate practices

Other drivers of indoor air pollution that emerged from the discussions were the prevailing practices with regard to ventilation. When asked about use of ventilation when cooking, it was clear that there was a disparity between knowledge and practice. For example, respondents shared that one needs to open the windows and doors when cooking with a kerosene or charcoal stove to vent the emissions, however, they reported that this was indeed not practised because of the poor nature of houses and the levels of insecurity in the communities. In addition, some houses were said to lack windows and people only opened the door during the day and had to endure the emissions at night as insecurity forces them to keep the door closed. Fear of the cold outdoor air was also mentioned as a reason why people chose not to open the windows or doors. It also emerged that space constraints prevented people from opening windows to let in fresh air.

In Korogocho, it emerged that fear of illnesses such as malaria and of the cold outdoor air drove people to negatively modify the eaves of their houses. When asked whether houses had eaves, several respondents answered in unison that *“many people fear malaria,”* and so to ward off mosquitoes and the cold, they chose to block the eaves with plastic bags and other materials available to them as indicated in these opinions:

*There is a space between the roof and wall but to prevent the cold, we have blocked it with gunny bags* (these are bags made of sisal or plastic fibre) *and curtains so there is no way air from in can get out or from outside get in”(Older female, Korogocho).*

… the ceiling is made of gunny bags, on the sides there are gunny bags so there are no spaces, so when smoke fills the room it just stays (older male, Korogocho).

#### Reducing outdoor and indoor air pollution

##### Resistance and ignorance

When asked what residents could do to reduce the levels of air pollution in the community, there were mixed reactions with some people feeling that there was nothing they could do. Fear of fights was cited as prohibiting people from asking neighbours or other community members to stop practices that were contributing to air pollution. On the other hand, there were those who felt that residents had a responsibility of ensuring the environment was kept clean, instead of waiting for outsiders to come and clean their backyard. Discussions about possible relocation of the dumpsite from Korogocho emerged to be unpopular among residents. Many residents rely on the dumpsite as a source of livelihood through scavenging; therefore, even though they were aware of the hazards the dumpsite posed, they were opposed to its relocation. As one participant put it:

See that dumpsite, there are groups [involved in scavenging for recyclable materials] and together with some priests we formed one environmental group, we started recycling. That dumpsite has created employment for youth; it has created employment for 30% and caused 5% deaths (Older male, Korogocho).

Similar opinions emerged in Viwandani where it was felt that relocation of industries to a less habited place would be resisted as many people were employed in the industries. It was also felt that such a move would be pointless as people would ‘follow’ the industries and continue living in close proximity to these industries. Also discussions around the residents’ opinions on temporary closure of the industries to allow installation of emission control systems seem to be an unpopular move due to its impact on jobs. However, participants agreed that there was need for the government to enforce strict emissions control measures in the industries.

Participants from both communities voiced the need to create awareness about air pollution and measures needed to reduce exposure as the following opinion indicates:

I think it would be good if people can be sensitized about the environment so that people know how their environment should be, the things they should do to avoid polluting or the effects of pollution; this will be achieved for example through discussions like this which has made us know where we were perhaps going wrong and we can correct that… If we had organizations that would sensitize people … So it is my appeal that for those who don’t know we have organizations that educate them on the environment so that people have that awareness to help reduce pollution (young male, Viwandani).

##### Fatalism and helplessness

There were sentiments of fatalism when asked whether residents were concerned about air pollution. The participants felt they could not do much to address the issue of air pollution. They appeared to have rationalized the state of the environment in which they lived and were resigned to it. Expressions indicating residents viewed their polluted space as normal were heard from both the young and older adults in both communities. One participant best summed it by saying, *“We are used… we have got used to this [air pollution] we forgot it is a problem” (Older female, Korogocho).*

In the discussions, there were those who felt helpless and they relied on religion to find solace as one participant indicated: *“It’s God who protects us, the kind of dirt we have seen in this place is a lot” (Young female, Korogocho)*. This sentiment also revealed a flawed state of knowledge on the health impacts of pollution and the lack of appreciation of the central role individuals ought to play in ensuring the environment is safe for them to live in.

There was a sense of frustration among respondents as they raised issues about the lack of a voice to petition leaders to address issues about the pollution occurring in their communities, especially, for those sources that were external to the slums. The Viwandani participants reported being threatened with eviction if they raised these issues. The following discussions reveal what residents faced:

We usually tell them and when we tell them, they mostly say we should know we are living under electric lines [high voltage lines] we will be moved. You hear a lot of things; we feel we are troubled; sewage line passes here, because we are not known by the government so we just decide to keep quiet so we can live here longer (Older male, Viwandani).

We tell them [government representatives] but we are told we should know that the government does not know there are people living here. We are forced to keep quiet but we know we are being oppressed (Older male, Viwandani).

On the other hand, Korogocho residents pointed fingers at the local leadership for worsening the pollution; for instance, allocating building space without leaving space for functions such as waste collection.

## Discussion and conclusion

This study explored the general perception and understanding among residents in two slums in Nairobi about air pollution and associated health risks in their community. We acknowledge that slum residents face various environmental challenges that are not limited to air pollution alone. However, since this study sought to assess the perceptions on air pollution, the discussion was limited to this objective. The main highlights indicate that residents of both slums relied on sensory perceptions to assess air pollution and its sources. There was an apparent disconnect between knowledge and practice as far as the use of ventilation was concerned. In addition, the participants expressed a lack of agency to address the current state of air quality in the two communities. These findings are similar to those from a recent quantitative study conducted in the two slums [23].

Outdoor air pollution in the two communities was mainly attributed to garbage dumpsites located in close proximity to both communities. Combustion of the garbage was cited to be a major contributor of smoke and soot in both locations, while industrial emissions were in addition, of great importance to Viwandani residents. Similar findings were observed by Howel and colleagues [24] on the important role of place in forming public perceptions. People’s perceptions on pollution were informed by their sensory experience, for instance, the visible clouds of smoke and soot from the dumpsites and factories as well as the odorous emissions from industries and drainage channels. Sensory perceptions have been reported as important in informing perceptions of sources and exposure [8,18,25] and in subsequent response to the exposure [25]. The reliance on sensory perceptions in these communities also raises a red-flag for pollutants that are not odorous. For example, indoor air pollution from carbon monoxide might be ignored with fatal consequences.

There was a feeling that many of the activities carried out by residents, such as burning trash, were of a scale that was smaller in importance as compared to the major polluters, namely the industries and dump sites. This shift of responsibility to other entities has been reported in other studies as a mechanism adopted by individuals to distance themselves from any direct contribution to the problem at hand [6]. We found no evidence of a ‘neighbourhood halo effect’ that has been reported in other studies, where people are unwilling to attribute pollution to their place of residence as compared to other areas [8,16,24,25]. This can be attributed to the loose attachment many slum residents have to their place of residence given the informal nature of the settlements and the obvious disadvantage in terms of access to services compared to the neighbouring middle and upper class residential areas. The absence of a ‘halo effect’ would be an important attribute in the event programs aimed at reducing air pollution or exposure, are introduced. This is because residents already identify their communities as polluted spaces; a fact that might make them more accepting of programs/interventions to address the issue. In addition, the findings of this study are contrary to the review by Saksena [5] that pointed to studies indicating that among people of low socio-economic status, air pollution was out-ranked by other more urgent issues. In fact, this study finds that as much as residents had other issues to think about, pollution was on the forefront as an issue of concern.

The feeling of helplessness can be attributed to the lack of voice among the residents to approach their leaders and to demand action. Given their informal residential status, poverty and lack of alternatives, many felt entrapped in this polluted space. It is also a consequence of poor social capital among residents, which inhibits collective action against pollution [16,26]. Residents raised the issue of counter threats whenever they went to the local leaders to petition them to take action against polluters. This helplessness was exacerbated by the lack of security of the tenure on the land on which residents lived and the informal status of the slums. Studies elsewhere have shown that lack of attachment to a place limits people’s investment in the place and can lead to inaction against pollution [8,16,18].

Further, there was an apparent lack of agency as people felt there was nothing they could do to reduce the levels of pollution in their communities; lack of agency has been attributed to lower socio-economic status [16,26]. This lack of agency could derail any efforts the government puts in place to address air pollution in these and similar communities. Therefore, the government must first address these barriers to ensure they effectively implement pollution control and other environmental protection policies.

People’s ignorance about the ‘true’ effects of air pollution was evident in their daily practices such as blocking the eaves to prevent cold, dust and mosquitoes. This finding could be explained by the lack of public education on issues concerning air pollution and indeed, environmental issues in general. In addition, people’s actions might be justified given the poor housing available in their communities and their lack of resources to facilitate residential moves to better houses. As such, residents had the hard choice of either letting in the cold, polluted outdoor air and mosquitoes or living with indoor air pollution.

This study has revealed the perceptions of slum residents regarding both indoor and outdoor air pollution. We find that only the first two pillars of the protection motivation theory informing this study are fulfilled in the study settings. However, the reported behaviour is not protective as it puts residents at higher risk of exposure to air pollution while lack of agency makes it difficult for residents to address air pollution. From the findings we conclude there is an urgent need to create awareness among residents on the effects of air pollution and the need for each individual to take part in reducing the levels of air and general environmental pollution.

However, the study faces some limitations on coverage and lack of inclusion of all stakeholders in the survey. The study covered only two slums and it would be useful to also understand the perceptions of residents in both formal and informal settlements. The current study interviewed people aged 18 years and above, however, it would be worthwhile to also to get the views of school going children particularly those aged 10–17 years. This age group represents a special group with high risk because of exposure to outdoor air pollution during outdoor activities. We also didn’t conduct key informant interviews which would have provided information on the ways to support the residents in reducing the health burden from air pollution. Despite these limitations the study still provides useful insights on the perceptions about air pollution among the urban poor residents.

### Future research and action

The following should be considered or included in future research as they were not considered in this study. First a similar study should be conducted in different parts of the city to enable a comparison of perceptions along socio-economic classes and across different neighbourhoods. Second, conduct a study to identify sustainable solutions to air pollution that can work not only in the study communities but city-wide and nationally by including key stakeholders. Third, conduct studies that include school-going children as participants.

## Competing interests

The authors declare there are no competing interests.

## Authors’ contributions

All authors conceptualized the study. KM and TE designed the study guide and conducted the FGDs. TE moderated the discussions while KM took notes, transcribed the discussions, analysed the data and drafted the manuscript. TE, NN, EK, NN reviewed all earlier drafts of the manuscript and all authors signed off the final version of the manuscript. All authors read and approved the final manuscript.

## Pre-publication history

The pre-publication history for this paper can be accessed here:

http://www.biomedcentral.com/1471-2458/14/226/prepub
